# A pilot to assess the feasibility and potential clinical utility of enhanced sleep management on inpatient wards in a mental health trust

**DOI:** 10.1192/bjo.2021.575

**Published:** 2021-06-18

**Authors:** Ambrina Roshi, Rose McGowan, Lauren Roberts, Stuart Watson, Kirstie Anderson, Patrick Keown, Rod Bowles, Ron Weddle, Sophie Connolly

**Affiliations:** 1 St Nicholas Hospital, Cumbria Northumberland Tyne Wear NHS Foundation Trust; 2 Newcastle University; 3 Cumbria, Northumberland, Tyne And Wear NHS Foundation Trust

## Abstract

**Aims:**

To assess the feasibility and utility of introducing the following changes on to in-patient units:

Structural and cultural adaptation to create a sleep friendly ward environment

A “Protected Sleep Time” between midnight and 6am

Routine screening for sleep disorders, including obstructive sleep apnoea and restless leg syndrome

**Background:**

Insomnia and other sleep disturbances are cause, correlate and consequence of psychiatric disorders. Routine hourly night time observations, ward noise, bright lights at night time, sleep disorders, insufficient exercise, insufficient day light exposure, too much caffeine and inappropriate psychotropic use are all causes of disturbed sleep (Horne 2018).

**Method:**

Seven wards participated in a pilot (SleepWell). These consisted of one male and two female Acute Wards (General Adult), a High Dependency Unit, a Neurorehabilitation ward, an in-patient dementia service and one rehabilitation ward. These wards were supported via an existing trust management structure and the pilot was specifically supported by two trust managers (RW and RB) and by a clinical director (PK). The expectation was that each ward would identify a sleep champion from existing staff to facilitate the changes. A “product” was developed which identified core sleep management features but, in addition, wards were not confined to these. The existing policy that all inpatients should be checked each hour over night was suspended for the pilot wards and the patients had protected sleep time (PST) if the MDT agreed that it was clinically appropriate.

Quantitative and qualitative techniques were used to identify facilitators of change, impact on sleep and, outcome.

**Result:**

Protected sleep was viewed positively by all staff and approximately 50% of patients on the pilot wards were able to have PST at some point in their admission. Routine sleep disorder assessments were harder to implement and 33% of patients were screened. There were no deaths or significant events on patients due to PST. Hypnotic use on the pilot wards reduced. It is anticipated that PST where it is safe will be rolled out across all adult and old age wards in the trust.

**Conclusion:**

With support, it has been feasible to change many aspects of sleep management across a breadth of inpatient units in a large NHS trust.

